# Sera from Remitting and Secondary Progressive Multiple Sclerosis Patients Disrupt the Blood-Brain Barrier

**DOI:** 10.1371/journal.pone.0092872

**Published:** 2014-03-31

**Authors:** Fumitaka Shimizu, Ayako Tasaki, Yasuteru Sano, Mihua Ju, Hideaki Nishihara, Mariko Oishi, Michiaki Koga, Motoharu Kawai, Takashi Kanda

**Affiliations:** Department of Neurology and Clinical Neuroscience, Yamaguchi University Graduate School of Medicine, Ube, Japan; Institute Biomedical Research August Pi Sunyer (IDIBAPS) - Hospital Clinic of Barcelona, Spain

## Abstract

**Background:**

Pathological destruction of blood-brain barrier (BBB) has been thought to be the initial key event in the process of developing multiple sclerosis (MS). The purpose of the present study was to clarify the possible molecular mechanisms responsible for the malfunction of BBB by sera from relapse-remitting MS (RRMS) and secondary progressive MS (SPMS) patients.

**Methods:**

We evaluated the effects of sera from the patients in the relapse phase of RRMS (RRMS-R), stable phase of RRMS (RRMS-S) and SPMS on the expression of tight junction proteins and vascular cell adhesion protein-1 (VCAM-1), and on the transendothelial electrical resistance (TEER) in human brain microvascular endothelial cells (BMECs).

**Results:**

Sera from the RRMS-R or SPMS patients decreased the claudin-5 protein expression and the TEER in BMECs. In RRMS-R, this effect was restored after adding an MMP inhibitor, and the MMP-2/9 secretion by BMECs was significantly increased after the application of patients' sera. In SPMS, the immunoglobulin G (IgG) purified from patients' sera also decreased the claudin-5 protein expression and the TEER in BMECs. The sera and purified IgG from all MS patients increased the VCAM-1 protein expression in BMECs.

**Conclusions:**

The up-regulation of autocrine MMP-2/9 by BMECs after exposure to sera from RRMS-R patients or the autoantibodies against BMECs from SPMS patients can compromise the BBB. Both RRMS-S and SPMS sera increased the VCAM-1 expression in the BBB, thus indicating that targeting the VCAM-1 in the BBB could represent a possible therapeutic strategy for even the stable phase of MS and SPMS.

## Introduction

Multiple sclerosis (MS) is defined as a chronic inflammatory demyelinating disease of the central nervous system, which is pathologically characterized by the presence of focal demyelinated plaques within the white matter [1]. MS is classified into three clinical subtypes including relapse-remitting MS (RRMS), primary progressive MS (PPMS) and secondary progressive MS (SPMS) [2]. The disease usually starts with a course of RRMS, which is eventually followed by a phase of SPMS in the majority of Caucasian patients [2]. On the other hand, both SPMS and PPMS are relatively rare in Japan. In RRMS, the pathological alterations in the brain are clearly associated with the inflammatory process, because newly formed lesions within the central nervous system (CNS) can be visualized by the contrast gadolinium (Gd)-enhancement of the brain parenchyma during magnetic resonance imaging (MRI), and anti-inflammatory therapies and immunomodulation exert a beneficial effect at this stage of the disease [3], [4]. In contrast, SPMS appears to be less driven by the inflammatory process than RRMS: Gd-enhancing lesions are rare, but progressive loss of brain volume is observed in MRI and, most importantly, the current immunomodulatory or anti-inflammatory treatments have little beneficial effect in SPMS [4]–[8].

Pathological breakdown of the BBB may be the early and prominent features of disease process in all clinical subtypes of MS [9]–[11]. Acute MS lesions have demonstrated disruption of the BBB, as evidenced by *in vivo* Gd-enhancement on MRI and post-mortem evidence of focal micro-vascular leakage [3], [12], [13]. In both PPMS and SPMS, persistent loss of BBB integrity, as indicated by vascular leakage and the disruption of tight junctions, has been clearly observed in active and inactive lesions [12]–[14]; however, the molecular mechanism underlying the breakdown of the BBB in each clinical subtype of MS has not been adequately explained.

There is accumulating evidence that disruptions of the BBB are mediated by some humoral factors including proinflammatory cytokines or matrix-metalloproteinase (MMP)-2/9 and these may be a crucial step in the pathogenesis of MS [15], [16] and experimental autoimmune encephalomyelitis (EAE) [17]. We thus hypothesized that humoral factors may be responsible for the disruption of the BBB in both RRMS and SPMS patients. The purposes of the current study were to ascertain whether the sera from patients with either RRMS or SPMS can disrupt the BBB, and to clarify the contributions of humoral factors in sera, particularly MMP-2/9 and antibodies against the human BBB-composing endothelial cells, to the malfunction of the BBB.

## Materials and Methods

### Sera

This study and the use of patients' sera were approved by the ethics committee of Yamaguchi University following the principles of the Declaration of Helsinki. All patients have provided their written informed consent to participate in this study. This consent procedure was also approved by the ethics committees of Yamaguchi University. The sera were collected from 23 MS patients who were diagnosed at Yamaguchi University Hospital. All patients met the clinical criteria based on the McDonald criteria [18]. Sera were obtained within one week after the first appearance of symptoms from six patients in the relapse phase of RRMS (RRMS-R), who presented with both new worsening of neurological symptoms associated with objective neurologic signs and the appearance of a new Gd-enhancing lesion in MRI. Nine RRMS patients in the stable phase (RRMS-S), who were being treated with IFN-β and had been in clinical remission for one year, were also enrolled in this study. The eight SPMS patients were defined as those with recently confirmed progression based on the Expanded Disability Status Scale (EDSS) score without relapse, so they had an EDSS>4. The sera from six healthy individuals served as normal controls. Blood samples were stored at −80^○^C until use. All sera were inactivated at 56^○^C for 30 minutes immediately prior to use.

### Cell culture and treatment

Immortalized human brain microvascular endothelial cells (BMECs), named “TY09”, were previously generated [19]. Cells were cultured in medium containing 10% patient serum or in culture medium containing 10% fetal bovine serum (FBS; Sigma, St. Louis, MO, U.S.A), which were applied as controls, in CO_2_ incubator at 37^○^C. The transendothelial electrical resistance (TEER) value was measured 24 hours later, and the total proteins were obtained the next day.

### Reagents

The culture medium for Cells was previously described [19]. Polyclonal anti-claudin-5 and anti-occludin antibodies were purchased from Zymed (San Francisco, CA, U.S.A). The polyclonal anti-actin, antibodies were obtained from Santa Cruz (Santa Cruz, CA, U.S.A). The polyclonal anti-TNF-α, anti-IFN-γ, anti-IL-17, anti-VEGF and anti- vascular cell adhesion molecule-1 (VCAM-1) antibodies were purchased from R&D systems (Minneapolis, MN, U.S.A).

### Western blot analysis

The equal amounts of protein (15 μg) for each sample were resolved by 10% SDS-PAGE (Biorad). After electrophoresis, the gels were transferred to PVDF membranes (Amersham, Chalfont, UK) as described previously [19]. The blots were incubated with relevant antibodies (dilution 1∶100) for 2 hours as the primary antibodies and then followed with a secondary antibody (dilution 1∶2,000) for 1 hour at room temperature. The bands were visualized by an enhanced chemiluminescence detection system (ECL-prime, Amersham, UK). Each band density was normalized by referring it to its actin band density using the Quantity One software program (Bio-Rad, Hercules, CA). The changes in the expression of tight junction proteins, including claudin-5 and occludin, and adhesion proteins, such as VCAM-1, in TY09 were examined following exposure to the patient's sera, as claudin-5 and occludin are now recognized to be key components involved in maintaining the properties of the BBB, and endothelial VCAM-1 as implicated in the transmigration of lymphocytes across the BBB [20]–[22].

### TEER studies

Cells were seeded on the Transwell inserts (pore size 0.4 μm, effective growth area 0.3 cm2, BD Bioscience, Sparks, MD, U.S.A) that were coated with rat-tail collagen type-I (BD Bioscience). The TEER values were measured by a Millicell electrical resistance apparatus (Endohm-6 and EVOM, World Precision Instruments, Sarasota, FL, U.S.A), as described previously [19]. Effects of each type of medium (non-conditioned medium used as a control, conditioned medium contained 10% patient sera) were evaluated as the change of TEER value. Resistances of blank filters were subtracted from those of filters with cells before final resistances were calculated.

### Quantitative analysis of the VEGF and MMP-2/9 by ELISA

Quantification of serum levels of VEGF and MMP-2/9 were assessed in triplicate by commercially available quantitative sandwich ELISA kits according to instructions of the manufacturers (R&D Systems, Minneapolis, MN). The results were expressed as picograms of VEGF per milliliter (pg/ml) or nanogram of MMP-2/9 per milliliter (ng/ml), based on the standards provided with the available kits.

### Treatment with neutralizing antibodies

The sera from MS patients were pre-treated with either a neutralizing antibody (2.0 μg/ml) against TNF-α, IFN-γ, IL-17, or VEGF, or were treated with normal rabbit IgG (control Ab) for six hours at 4°C. Cells were cultured with the patients' sera containing each neutralizing antibody at 37°C.

### Treatment with a MMP inhibitor

A broad-spectrum MMPs inhibitor, GM6001 (Chemicom, Temecula, CA, USA) was prepared for the inhibition study. The sera from MS patients were pre-treated with 25 μM of GM6001 for 12 hour at 37°C. Cells were cultured with the sera from MS patients with or without GM6001.

### IgG purification and exposure to purified IgG

The IgG fractions were obtained from the sera of patients or five healthy individuals by affinity chromatography using a Melon™ Gel IgG Spin Purification Kit (Thermo Scientific, Rockford, IL, U.S.A). Cells were treated with culture medium containing either purified patient or healthy individual IgG (final concentration 400 μg/mL). Cells treated with culture medium containing purified IgG obtained from FBS (Sigma, final concentration 400 μg/mL) were used as controls.

### Data analysis

All data are presented as the means ± SEM. The values were compared using the analysis of variance followed two-tailed Student's *t*-tests. A P value of less than 0.05 was considered to be statistically significant.

## Results

### The sera from relapsing MS or SPMS patients reduced the expression of tight junction molecules, and the sera from all clinical subtypes of MS patients increased the amount of VCAM-1 in TY09 cells

**Figure 1:**
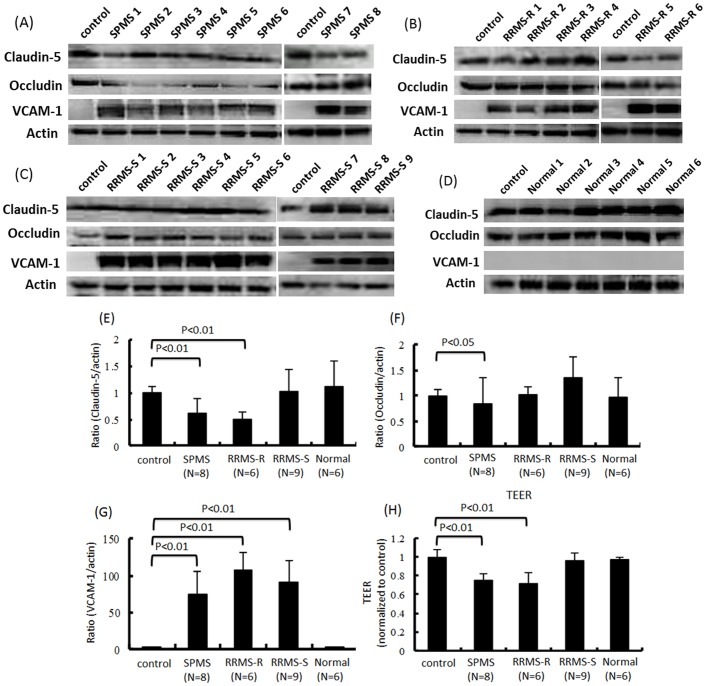
The effects of MS patients' sera on the tight junction proteins and adhesion molecules in TY09 cells. (A)–(D) The changes in the amounts of claudin-5, occludin and VCAM-1 in human brain microvascular endothelial cells, named “TY09 cells”, were determined after exposure to the sera from patients in the relapse phase of MS (RRMS-R), the stable phase of RRMS (RRMS-S) or secondary progressive MS (SPMS), or from healthy controls, as determined by a Western blot analysis. (E)(F)(G) Each bar graph reflects the combined densitometry data from each independent experiment (mean ± SEM, SPMS n = 8, RRMS-R n = 6, RRMS-S n = 9, Normal n = 6). (E)(F) The amount of claudin-5 protein in TY09 cells was significantly decreased after the exposure to sera from SPMS or RRMS-R patients, and the amount of occludin protein in TY09 cells was significantly reduced after exposure to sera from SPMS patients, whereas the amounts of the claudin-5 and occludin proteins were not significantly affected by exposure to sera from RRMS-S patients or healthy controls. (G) The amount of VCAM-1 was significantly increased after exposure to sera from RRMS-R, RRMS-S or SPMS patients (H) The TEER value of BMECs was significantly decreased after exposure to RRMS-R or SPMS sera, but was not influenced by exposure to sera from RRMS-S patients or healthy controls (mean±SEM, SPMS n = 8, RRMS-R n = 6, RRMS-S n = 9, Normal n = 6). Control: non-conditioned DMEM containing 20% FBS; SPMS: conditioned medium with 10% serum from an SPMS patient diluted with non-conditioned DMEM containing 10% FBS; RRMS-R: conditioned medium with a 10% concentration of serum from an RRMS-R patient diluted with non-conditioned DMEM containing 10% FBS; RRMS-S: conditioned medium with a 10% concentration of serum from an RRMS-S patient diluted with non-conditioned DMEM containing 10% FBS; Normal: conditioned medium with 10% serum from a healthy control diluted with non-conditioned medium of DMEM containing 10% FBS.

We first examined the effects of sera from each clinical subtype of patients, including RRMS-R, RRMS-S and SPMS patients, on the expression of tight junction proteins and adhesion molecules in TY09. The amount of claudin-5 in TY09 was significantly decreased after exposure to sera from patients with RRMS-R or SPMS, whereas it was not affected by the sera from RRMS-S patients or from healthy controls, as determined by a Western blot analysis (**Figs. 1A–F**). The amount of occludin protein in TY09 was also significantly decreased after exposure to the sera from SPMS patients, although it was not changed after exposure to sera from RRMS-R or RRMS-S patients, or from healthy controls (**Figs. 1A–F**). In contrast, the amount of VCAM-1 protein in TY09 was significantly increased after exposure to sera from all MS patients, whereas it was not changed by the sera from healthy controls (**Figs. 1A–D, G**). The TEER value of TY09 was significantly decreased after exposure to sera from RRMS-R or SPMS patients, although it was not changed by incubation with sera from RRMS-S patients or from healthy controls (**Fig. 1H**).

### Purified serum IgG from SPMS patients reduced the amount of claudin-5 protein, and IgG from all clinical subtypes of MS patients increased the amount of VCAM-1 protein in TY09 cultures

**Figure 2:**
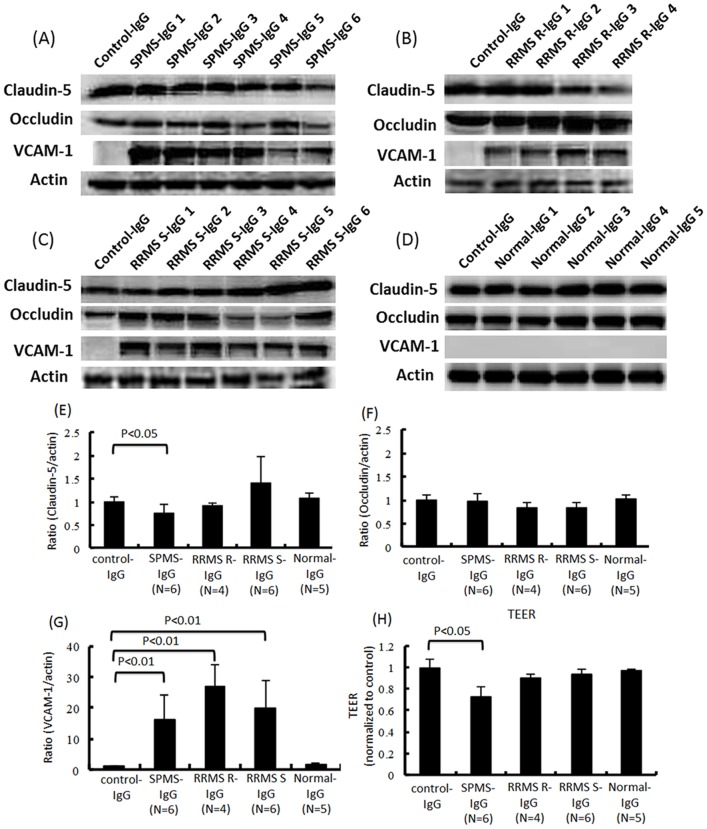
Purified serum IgG from SPMS patients decreased the amount of claudin-5 protein, and IgG from all clinical subtypes of MS patients increased the amount of VCAM-1 protein in TY09 cells. (A)–(D) The effects of the purified serum IgG from MS patients on the expression of claudin-5, occludin, and VCAM-1 in TY09 cells was determined by a Western blot analysis. (E)(F)(G) Each bar graph reflects the combined densitometry data from independent experiments (mean ± SEM, SPMS n = 6, RRMS-R n = 4, RRMS-S n = 6, Normal n = 5). (E) The amount of claudin-5 protein in the TY09 cells was significantly decreased after exposure to the purified serum IgG fractions of SPMS patients, whereas it was not affected by the purified IgG fractions from RRMS-R or RRMS-S patients, or from healthy controls, as determined by a Western blot analysis. (F) The amount of occludin was not significantly affected by exposure to the purified IgG fractions from all of the MS patients or from healthy controls. (G) The amount of VCAM-1 protein in the TY09 cells was significantly increased after exposure to the purified IgG fraction from patinets with all clinical subtypes of MS, whereas it was not changed by the IgG from healthy controls, as determined by a Western blot analysis. (H) The TEER value of the TY09 cells was significantly decreased after exposure to the purified IgG fraction from SPMS patients, although it was not changed by incubation with the purified serum IgG fractions from RRMS-R or RRMS-S patients, or from healthy controls (mean ± SEM, SPMS n = 6, RRMS-R n = 4, RRMS-S n = 6, Normal n = 5). Control-IgG: conditioned medium containing purified IgG fractions obtained from FBS; SPMS-IgG: conditioned medium containing purified IgG fractions obtained from the sera of SPMS patients; RRMS R-IgG: conditioned medium containing purified IgG fractions obtained from the sera of RRMS-R patients; RRMS S-IgG: conditioned medium containing purified IgG fractions obtained from the sera of RRMS-S patients; Normal-IgG: conditioned medium containing purified IgG fractions obtained from the sera of healthy individuals.

We next examined whether the purified serum IgG from patients with each clinical subtype of MS was responsible for the disruption of the BBB. The amount of claudin-5 in TY09 was significantly decreased after exposure to the purified IgG fractions of sera from SPMS patients, whereas it was not affected by those from patients with RRMS-R, RRMS-S or from healthy controls, as determined by a Western blot analysis (**Figs. 2A–D, E**). The amount of occludin was not changed after a challenge with the serum IgG from MS patients and healthy controls (**Figs. 2A–D, F**). The TEER value of TY09 was significantly decreased after exposure to the purified IgG fractions from SPMS patients, but was not changed by incubation with those from RRMS-R, RRMS-S or healthy control patients (**Fig. 2H**). In addition, the amount of VCAM-1 protein in TY09 was significantly increased after exposure to the purified IgG fraction from all clinical subtypes of MS patients, whereas it was not changed by those from healthy controls, as determined by a Western blot analysis (**Figs. 2G**). We next analyzed whether specific autoantibodies against human BMECs were present in the purified IgG fractions of the SPMS patients' sera by a Western blot analysis. Antibodies that bound to TY09 were detected in the purified IgG fractions from the MS patients' sera, which predominantly reacted with one or more antigens of approximately 10, 22, 28, 32, 38, 40, 50 and 60 kDa in TY09 lysates (**Fig. S1**). However, no specific bands for SPMS patients were detected.

### The sera from relapsing MS disrupted the BBB through the up-regulation of autocrine MMP-2/9 secretion in TY09 cells

**Figure 3:**
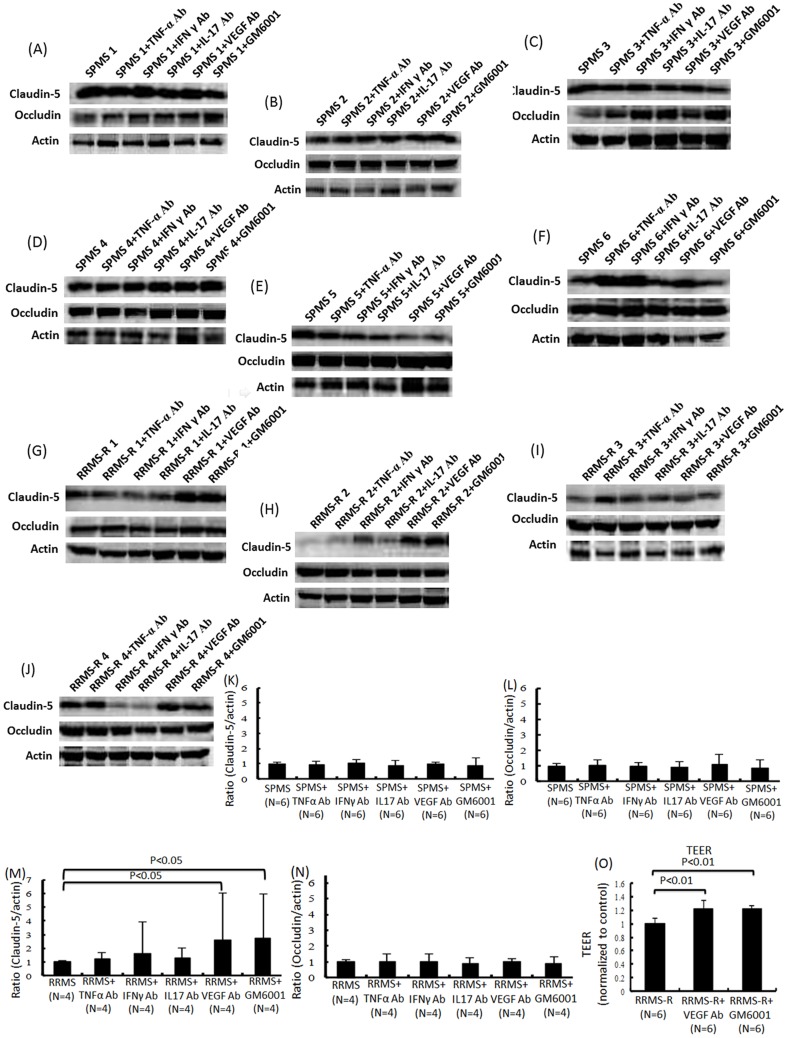
The BBB disruption was restored after adding GM6001 or a neutralizing anti-VEGF antibody to the sera from relapsing MS patients. (A)–(J) The effects of TNF-α, IFN-γ, IL-17 or VEGF neutralizing antibodies or a matrix- metalloproteinases (MMPs) inhibitor, GM6001, on the amount of tight junction proteins in TY09 cells after exposure to the sera from SPMS or RRMS-R patients was determined by a Western blot analysis. (K)–(N) Each bar graph reflects the combined densitometry data from each independent experiment (mean±SEM, SPMS n = 6, RRMS-R n = 4). (M) In patients with RRMS-R, preincubation with a VEGF neutralizing antibody or GM6001 increased the amount of claudin-5 protein in TY09 cells. Part 2 (O) The TEER value of the TY09 cells significantly increased after incubation with the sera from RRMS-R patients that had been pretreated with an anti-VEGF neutralizing antibody or GM 6001 (mean±SEM, n = 6). SPMS: conditioned medium containing a 10% concentration of serum from a SPMS patient diluted with non-conditioned DMEM containing 10% FBS; SPMS+TNF-α Ab: conditioned medium with 10% SPMS sera pretreated with a TNF-α neutralizing antibody; SPMS+IFN-γ Ab: conditioned medium with 10% SPMS sera pretreated with an IFN-γ neutralizing antibody; SPMS+IL-17 Ab: conditioned medium with 10% SPMS sera pretreated with an IL-17 neutralizing antibody; SPMS+VEGF Ab: conditioned medium with 10% SPMS sera pretreated with a VEGF neutralizing antibody; SPMS+GM6001: conditioned medium with 10% SPMS sera pretreated with GM6001; RRMS-R: conditioned medium containing a 10% concentration of serum from a RRMS-R patient diluted with non-conditioned DMEM containing 10% FBS; RRMS-R+TNF-α Ab: conditioned medium with 10% RRMS-R sera pretreated with a TNF-α neutralizing antibody; RRMS-R+IFN-γ Ab: conditioned medium with 10% RRMS-R sera pretreated with an IFN-γ neutralizing antibody; RRMS-R+IL-17 Ab: conditioned medium with 10% RRMS-R sera pretreated with an IL-17 neutralizing antibody; RRMS-R+VEGF Ab: conditioned medium with 10% RRMS-R sera pretreated with a VEGF neutralizing antibody; RRMS-R+GM6001: conditioned medium with 10% RRMS-R sera pretreated with GM6001.

**Figure 4:**
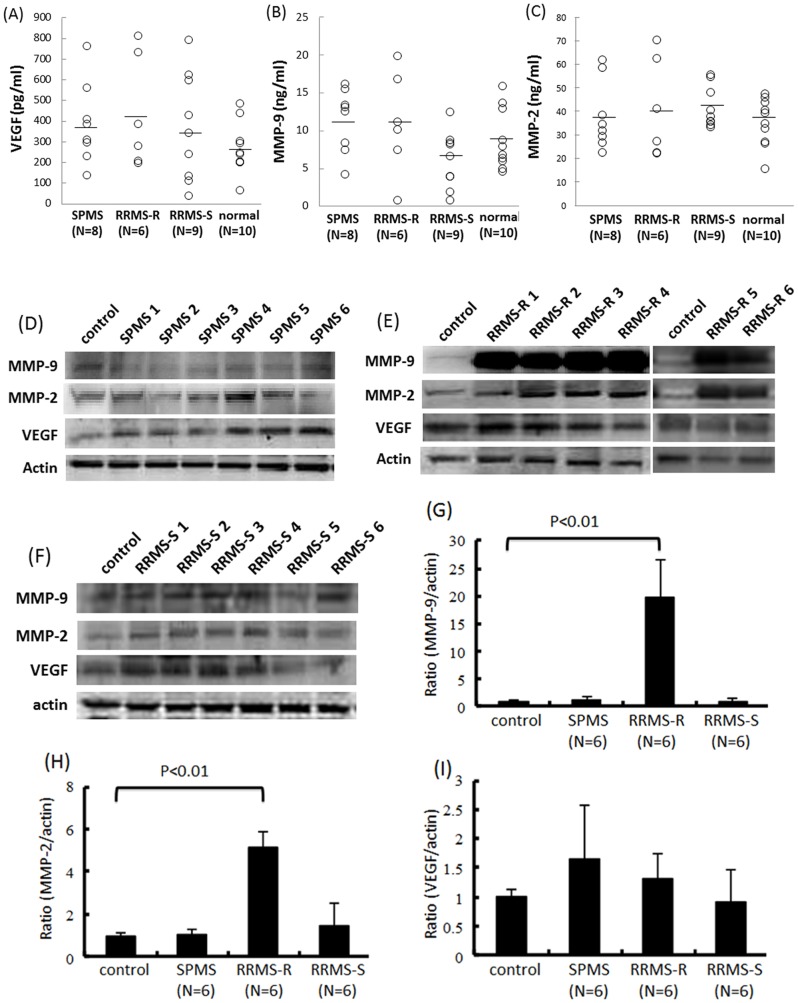
The sera from relapsing MS increased the autocrine MMP-2/9 secretion in TY09 cells. (A)–(C) The VEGF or MMP-2/9 concentrations were analyzed in the sera from patients with SPMS, RRMS-R or RRMS-S, or from healthy control subjects. The bars indicate the mean of each group. No significant differences were observed between the four groups. (D)–(F) The amounts of VEGF or MMP-2/9 by TY09 cells after exposure to sera from SPMS, RRMS-R or RRMS-S patients. (G)–(I) Each bar graph reflects the combined densitometry data from each independent experiment (mean ± SEM, SPMS n = 6, RRMS-R n = 6, RRMS-S n = 6). The amounts of MMP-2 and MMP-9 protein in the TY09 cells were significantly increased after exposure to the sera from RRMS-R patients, although it did not change after exposure to the sera from SPMS or RRMS-S patients. Control: non-conditioned DMEM containing 20% FBS; SPMS: conditioned medium with 10% serum from an SPMS patient diluted with non-conditioned DMEM containing 10% FBS; RRMS-R: conditioned medium with a 10% concentration of serum from an RRMS-R patient diluted with non-conditioned DMEM containing 10% FBS; RRMS-S: conditioned medium with a 10% concentration of serum from an RRMS-S patient diluted with non-conditioned DMEM containing 10% FBS.

To elucidate the contribution of inflammatory cytokines and MMP-2/9 to the BBB breakdown in patients with RRMS-R or SPMS, the TNF-α, IFN-γ, IL-17 or VEGF activities were neutralized using the corresponding neutralizing antibodies. MMP-2/9 were also inhibited by the broad-spectrum MMPs inhibitor, GM6001 (**Figs. 3 A–J**). The amount of claudin-5 protein in TY09 significantly increased after exposure to the RRMS-R sera pretreated with the anti-VEGF neutralizing antibody or GM6001, as determined by a Western blot analysis (**Figs. 3 M**), whereas it did not change after pre-incubation with TNF-α, IFN-γ or IL-17 neutralizing antibodies or after exposure to the SPMS sera pretreated with the neutralizing antibodies or GM6001 (**Figs. 3 K, L**). The TEER value of TY09 also significantly increased after exposure to RRMS-R sera pretreated with the anti-VEGF antibody or GM6001 (**Figs. 3 O**). The serum concentrations of VEGF and MMP-2/9 did not significantly differ among the different phenotypes of MS patients and healthy controls, as determined by an ELISA-based method (**Figs. 4A–C**). We thus considered that RRMS-R sera may disrupt the BBB by increasing the autocrine secretion of VEGF or MMP-2/9. The amount of MMP-2/9 in TY09 was found to be significantly increased after exposure to sera from RRMS-R patients while it did not change after exposure to the sera from RRMS-S, or SPMS patients (**Figs. 4 D–I**). The amount of VEGF was not changed after the exposure to sera from MS patients (**Figs. 4 D–I**).

## Discussion

The disruption of the BBB and the degradation of tight junction proteins at the BBB may play a significant role in the disease process in all three clinical subtypes of MS (RRMS, PPMS and SPMS). Minagar et al. demonstrated that the serum from relapsing MS patients containing elevated levels of pro-inflammatory cytokines decreased the occludin expression in cultured endothelial cells [23]. The pathological findings using autopsy brain section from patients with SPMS or relapsing MS demonstrated that the highest level of BBB disruption including a lack of ZO-1 and occludin was observed in active lesions (affecting 40% of vessels), although it was more apparent in inactive lesions (23%) and normal-appearing white matter (NAWM) (13%) than in neurological (8%) and normal (4%) controls [12], [13]. This result also indicates that a leaky BBB in the relapsing MS or SPMS patients is observed not only in active lesions, but also in inactive lesions or NAWN. However, Gd-enhanced lesions in MRI studies are rarely observed in SPMS patients. This discrepancy may come from the fact that the mild BBB disturbance appears to be below the detection limit for contrast Gd-enhancement in MRI, but was seen around many brain vessels, even in completely pathologically inactive plaques [7], [8]. Furthermore, some evidence has suggested that humoral factors in MS sera disrupt the BBB. For example, a leaky BBB is seen in active lesions of relapsing MS, not only in inflamed vessels, but also in adjacent vessels without perivascular inflammatory cells [8], [24], suggesting that humoral factors by themselves can impair BBB permeability. We thus hypothesized that humoral factors in MS sera, especially sera from relapsing MS or SPMS patients, disrupt the BBB, and that the phenotypic discrepancy between the remitting phase of RRMS and SPMS may be derived from the differences in BBB breakdown. Namely, SPMS sera can cause mild but diffuse BBB disruption, thus inducing progressive exacerbation in the clinical symptoms without relapse, whereas remission phase sera in RRMS patients cannot disrupt the BBB. In the present study, we used conditionally-immortalized human BBB-derived endothelial cells (TY09) to analyze the effects of sera from MS patients on the impairment of the BBB function [19]. Our present study demonstrated that the sera from patients with relapsing MS or SPMS can induce the BBB breakdown. The expression of claudin-5 and the TEER values were decreased in TY09 after exposure to relapsing MS or SPMS sera. The amount of occludin protein was also decreased, but only after incubation with SPMS sera. Together, these results indicate that humoral factors in relapsing MS or SPMS sera disrupt the BBB, but those in the sera from MS patients in the remission phase do not influence the BBB. This finding supports our hypothesis concerning the BBB disturbance in MS: humoral factors in the sera from both relapsing MS and SPMS patients disrupt the BBB, and the mild BBB disturbance may be associated with the development of progressive stage of the disease in SPMS patients. We previously demonstrated that sera from the acute phase of MS did not influence the amount of claudin-5 or the TEER [25]. This discrepancy between our previous study and present results may be derived from the different criteria used to define MS relapse; in the present study, we enrolled MS patients in the acute phase of relapse who presented with both new exacerbating neurological symptoms with objective neurologic signs and the presence of a new Gd-enhanced lesion in MRI.

We next tried to identify the most important substance involved in disrupting the BBB in relapsing MS or SPMS patients. Recent data suggest that VEGF was able to induce BBB impairment [26], [27], and an increase in serum VEGF concentration might be involved in MS relapse [28]. In addition, the elevation of the serum MMP-2/9 concentration was also observed in relapsing MS [27], and the MMP-2/9-induced breakdown of the BBB has been suggested to be involved in the pathogenesis of MS and EAE [17], [29]. Our present study demonstrated that the BBB function was restored after adding GM6001 or a neutralizing anti-VEGF antibody to the sera from relapsing MS patients, indicating that MMP-2/9 and VEGF are the key molecules responsible for the disruption of the BBB in the relapsing MS patients. The serum concentration of MMP-2/9 or VEGF was not significantly increased in the relapsing MS patients compared to that from the sera from patients in the remission phase of MS, SPMS or from healthy controls; however, the secretion of MMP-2/9 by TY09 was increased after exposure to relapsing MS sera, although that of VEGF was not changed. This finding suggests that the effects of MMP-2/9 occurred due to an autocrine mechanism, thus, minimal secretion which does not influence the serum concentration may still lead to a significant effect. The results showing that the secretion of VEGF by TY09 was not influenced after exposure to sera from relapsing MS patients does not conflict with our hypothesis, in which endothelial cells secret VEGF in relapsing MS in a very small amount that might be sufficient for the destruction of BBB but insufficient to increase the serum concentration, or to be detected as the significant difference by a Western blot analysis. However, a larger cohort study regarding whether the serum concentrations of MMP-2/9 and VEGF in MS patients are increased is required before appropriate conclusions can be drawn. Our present studies also demonstrated that GM6001 may have therapeutic potential for repairing BBB damage in MS patients with disease relapse. A previous report suggested that treatment of GM6001 ameliorates EAE by repairing the disruption of the BBB [30], while, the drug can induce undesirable side effects at the clinical use because it is probably not sufficiently selective. Novel treatment to manipulate the BBB using more selective MMP inhibitors in the acute stage of the disease might therefore become a promising therapeutic approach for MS relapse.

We next hypothesized that antibodies binding to BMECs might be involved in the BBB disruption in MS patients, because our previous report showed that antibodies against BMECs were found in the sera from MS patients [25]. We thus determined whether purified serum IgG from patients with each clinical subtype of MS would have a direct influence on the BBB properties. Our results demonstrated that the purified IgG from SPMS sera decreased the amount of claudin-5 and the TEER of the BBB, thus indicating that unknown IgG antibodies against BMECs from SPMS sera may cause the disruption of the BBB. We speculate that the antibody-mediated BBB disruption in SPMS patients may cause a penetration of circulating neurotoxic substances across the BMECs, thus inducing the neurodegenerative phase of SPMS [31], [32]. This is supported by the fact that subtle dysfunction of the BBB is implicated in the pathogenesis of several neurodegenerative diseases including Alzheimer's disease and amyotrophic lateral sclerosis [33].

VCAM-1 has been shown to be expressed in MS lesion, although it is rarely detected in normal brain tissue [34]. Our results have demonstrated that the sera and purified IgG from all clinical subtypes of MS increased the amount of VCAM-1 protein in TY09. Natalizumab is a humanized monoclonal antibody against α4-integrins that is approved for treating RRMS. It inhibits the binding of leukocytes to the VCAM-1 expressed on activated brain vessels [22], [35], [36]. Cadavid, et al. recently reported that natalizumab may have efficacy even in patient with SPMS [37]. Our present results provide the theoretical basis for applying natalizumab even for patients with the stable phase of RRMS or SPMS, and indicate that therapy directed specifically towards the reduction of VCAM-1 in the BBB could be a possible new strategy for the active or stable phase of MS or SPMS.

In conclusion, our study demonstrated that the disruption of the BBB caused by the humoral factors present in the sera of MS patients involves two differentially regulated mechanisms: one is the disruption of BBB function via the autocrine secretion of MMP-2/9 from BMECs in relapsing MS or the exposure to autoantibodies against BMECs in SPMS, and the other is the up-regulation of VCAM-1 in BMECs by exposure to autoantibodies against BMECs present in MS sera. These data may provide novel explanations concerning the phenotypic differences between the remitting phase of MS and SPMS: the disruption of the BBB by SPMS sera is necessary for the development of progressive stage of the disease. A further understanding of the molecular mechanisms underlying the BBB breakdown in MS would lead to the development of therapies for this severe disabling disease.

## Supporting Information

Figure S1 **Representative results obtained by immunoblotting of the TY09 lysates.** The blots were exposed to the purified serum IgG from RRMS-R (n = 4), RRMS-S (n = 6) or SPMS (n = 6) patients, or from healthy controls (n = 5) after a total of 20 mg of protein lysates from TY09 were loaded. The purified IgG fractions of MS patients' sera predominantly reacted with one or more antigens of approximately 10, 22, 28, 32, 38, 40, 50 or 60 kDa in the TY09 lysates. However, no specific bands for SPMS patients were detected. RRMS R-IgG: conditioned medium containing purified IgG fractions obtained from the sera of RRMS-R patients; RRMS S-IgG: conditioned medium containing purified IgG fractions obtained from the sera of RRMS-S patients; Normal-IgG: conditioned medium containing purified IgG fractions obtained from the sera of healthy individuals.
(TIF)
