# Supplementary figures and images for: Sera from Remitting and Secondary Progressive Multiple Sclerosis Patients Disrupt the Blood-Brain Barrier
Source: PLoS One. 2014 Mar 31;9(3):e92872. doi: 10.1371/journal.pone.0092872 (PMC3970956; doi:10.1371/journal.pone.0092872)

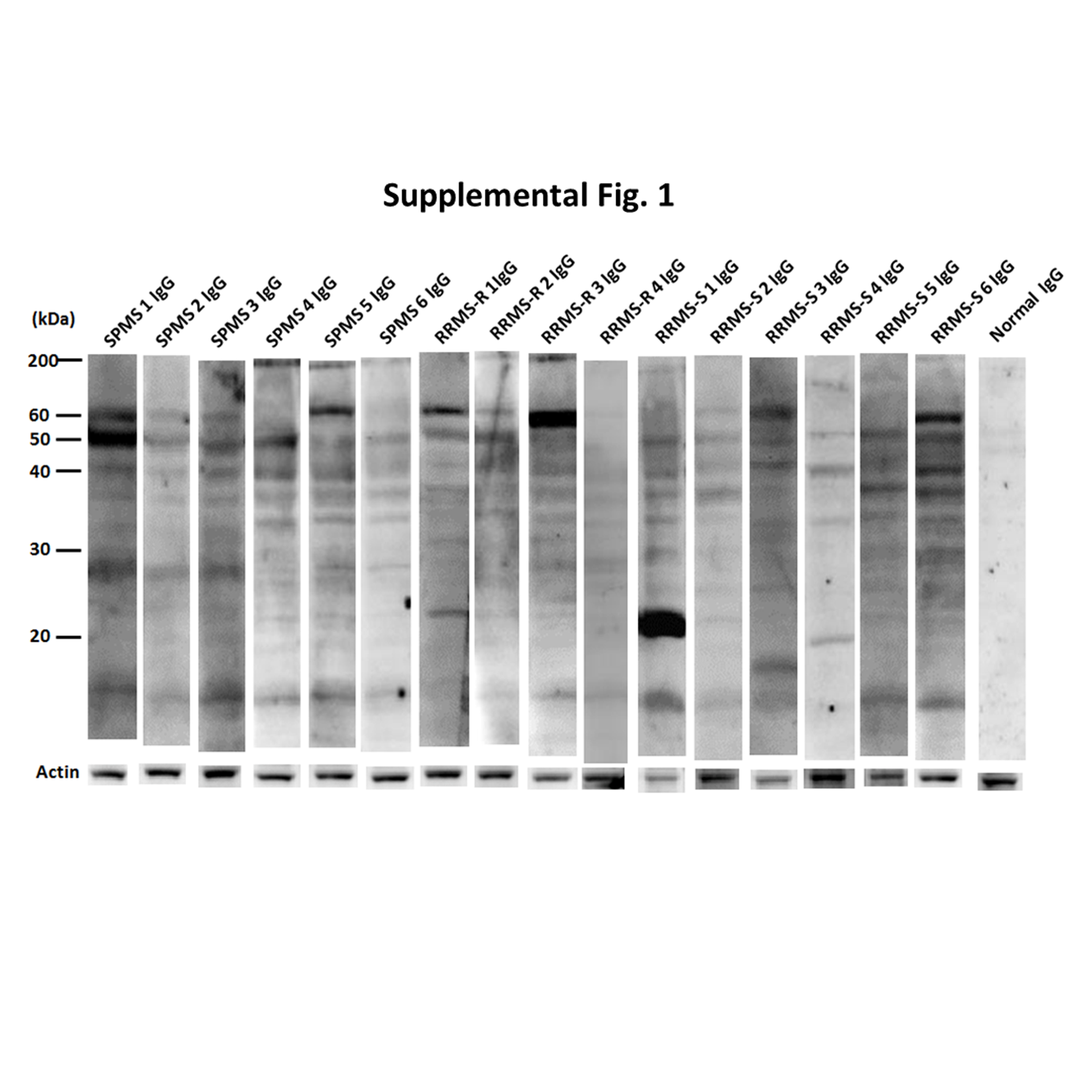

Supplement: Figure S1 — Representative results obtained by immunoblotting of the TY09 lysates. The blots were exposed to the purified serum IgG from RRMS-R (n = 4), RRMS-S (n = 6) or SPMS (n = 6) patients, or from healthy controls (n = 5) after a total of 20 mg of protein lysates from TY09 were loaded. The purified IgG fractions of MS patients' sera predominantly reacted with one or more antigens of approximately 10, 22, 28, 32, 38, 40, 50 or 60 kDa in the TY09 lysates. However, no specific bands for SPMS patients were detected. RRMS R-IgG: conditioned medium containing purified IgG fractions obtained from the sera of RRMS-R patients; RRMS S-IgG: conditioned medium containing purified IgG fractions obtained from the sera of RRMS-S patients; Normal-IgG: conditioned medium containing purified IgG fractions obtained from the sera of healthy individuals. (TIF) [file pone.0092872.s001.tif]
